# Absence of Aquaporin-4 in Skeletal Muscle Alters Proteins Involved in Bioenergetic Pathways and Calcium Handling

**DOI:** 10.1371/journal.pone.0019225

**Published:** 2011-04-28

**Authors:** Davide Basco, Grazia Paola Nicchia, Angelo D'Alessandro, Lello Zolla, Maria Svelto, Antonio Frigeri

**Affiliations:** 1 Department of General and Environmental Physiology, University of Bari-Aldo Moro, Bari, Italy; 2 Centre of Excellence in Comparative Genomics (CEGBA), University of Bari-Aldo Moro, Bari, Italy; 3 Department of Environmental Sciences, Tuscia University, Largo dell'Università snc, Viterbo, Italy; Instituto Nacional de Câncer, Brazil

## Abstract

Aquaporin-4 (AQP4) is a water channel expressed at the sarcolemma of fast-twitch skeletal muscle fibers, whose expression is altered in several forms of muscular dystrophies. However, little is known concerning the physiological role of AQP4 in skeletal muscle and its functional and structural interaction with skeletal muscle proteome. Using AQP4-null mice, we analyzed the effect of the absence of AQP4 on the morphology and protein composition of sarcolemma as well as on the whole skeletal muscle proteome. Immunofluorescence analysis showed that the absence of AQP4 did not perturb the expression and cellular localization of the dystrophin-glycoprotein complex proteins, aside from those belonging to the extracellular matrix, and no alteration was found in sarcolemma integrity by dye extravasation assay. With the use of a 2DE-approach (BN/SDS-PAGE), protein maps revealed that in quadriceps, out of 300 Coomassie-blue detected and matched spots, 19 proteins exhibited changed expression in AQP4^−/−^ compared to WT mice. In particular, comparison of the protein profiles revealed 12 up- and 7 down-regulated protein spots in AQP4−/− muscle. Protein identification by MS revealed that the perturbed expression pattern belongs to proteins involved in energy metabolism (i.e. GAPDH, creatine kinase), as well as in Ca^2+^ handling (i.e. parvalbumin, SERCA1). Western blot analysis, performed on some significantly changed proteins, validated the 2D results. Together these findings suggest AQP4 as a novel determinant in the regulation of skeletal muscle metabolism and better define the role of this water channel in skeletal muscle physiology.

## Introduction

Aquaporin-4 (AQP4) is the most important water channel of the neuromuscular system. AQP4 is expressed in skeletal muscle plasmalemma [1], and in particular in fast-twitch fibers [2], in which it determines increased water permeability [2]. In a previous work we hypothesized that AQP4, together with the endothelial AQP1, allows high water exchange between the blood and the fibers in order to regulate volume changes occurring during muscle activity [2] which may be related to the substantial muscle swelling and intracellular osmolyte production occurring during exercise [3]–[5]. The physiological relevance of this water channel in the skeletal muscle is supported by the fact that muscle activity modulates AQP4 expression as it is evident during disuse [6], [7].

Importantly, AQP4 expression is compromised in several muscle diseases, such as in hereditary muscular dystrophies, in which components of DGC are lost or strongly altered. For example, AQP4 is strongly reduced in skeletal muscle of Duchenne muscular dystrophy (DMD) patients [8]–[11] as well as in the sarcolemma of the mdx mouse, an animal model of the disease [2], [8], [9], [12]. Moreover, AQP4 down-regulation was observed in human patients affected by Limb Girdle Muscular Dystrophies (LGMDs) [13], in which defects in several isoforms of sarcoglycan (SG) occur. Reduction of AQP4 expression has often been associated to a marked reduction in α1-syntrophin (α1-syn) level, because of the close association between them [14], [15]. All these findings supported the idea that AQP4 water channels may be associated to dystrophin-glycoprotein complex (DGC).

In order to gain better insight into the role of AQP4 in skeletal muscle, we took advantage of the AQP4-null mouse model [16]. Previous studies performed on the same murine model have shown that AQP4 plays a pivotal role in modulating astrocytic function [17], [18] and preserving the blood brain barrier [19]. Furthermore, AQP4 might be involved in the proliferation, survival, migration and neuronal differentiation of adult neural stem cells (ANSCs) [20]. However, no investigations have been conducted in skeletal muscle regarding the effect of the absence of AQP4 on DGC and other sarcolemma proteins. Thus, the main purpose of the present study was: a) to evaluate the effect that the lack of AQP4 has on DGC and ECM expression and localization; and b) to conduct a more global protein analysis to assess altered protein patterns in skeletal muscle of AQP4-null mice.

The results presented in this study contribute to a better understanding of the relationship between AQP4 water channel, the DGC and the ECM, and suggest potential new physiological roles of this aquaporin in skeletal muscle activity.

## Results

### DGC expression analysis by immunofluorescence

Several studies have reported that AQP4 is markedly decreased in muscle fibers from myopathies in which DGC protein expression is severely compromised, such as DMD and LGMDs [8], [9], [13]. To investigate whether the absence of AQP4 affects membrane association and expression of DGC components, indirect immunofluorescence was performed on transverse cryosections of WT and AQP4^−/−^ quadriceps muscle, a typical example of fast-twitch fiber skeletal muscle. In addition to the core components of the DGC, levels of dysferlin and caveolin-3 (cav-3) were also analyzed.

**Figure 1:**
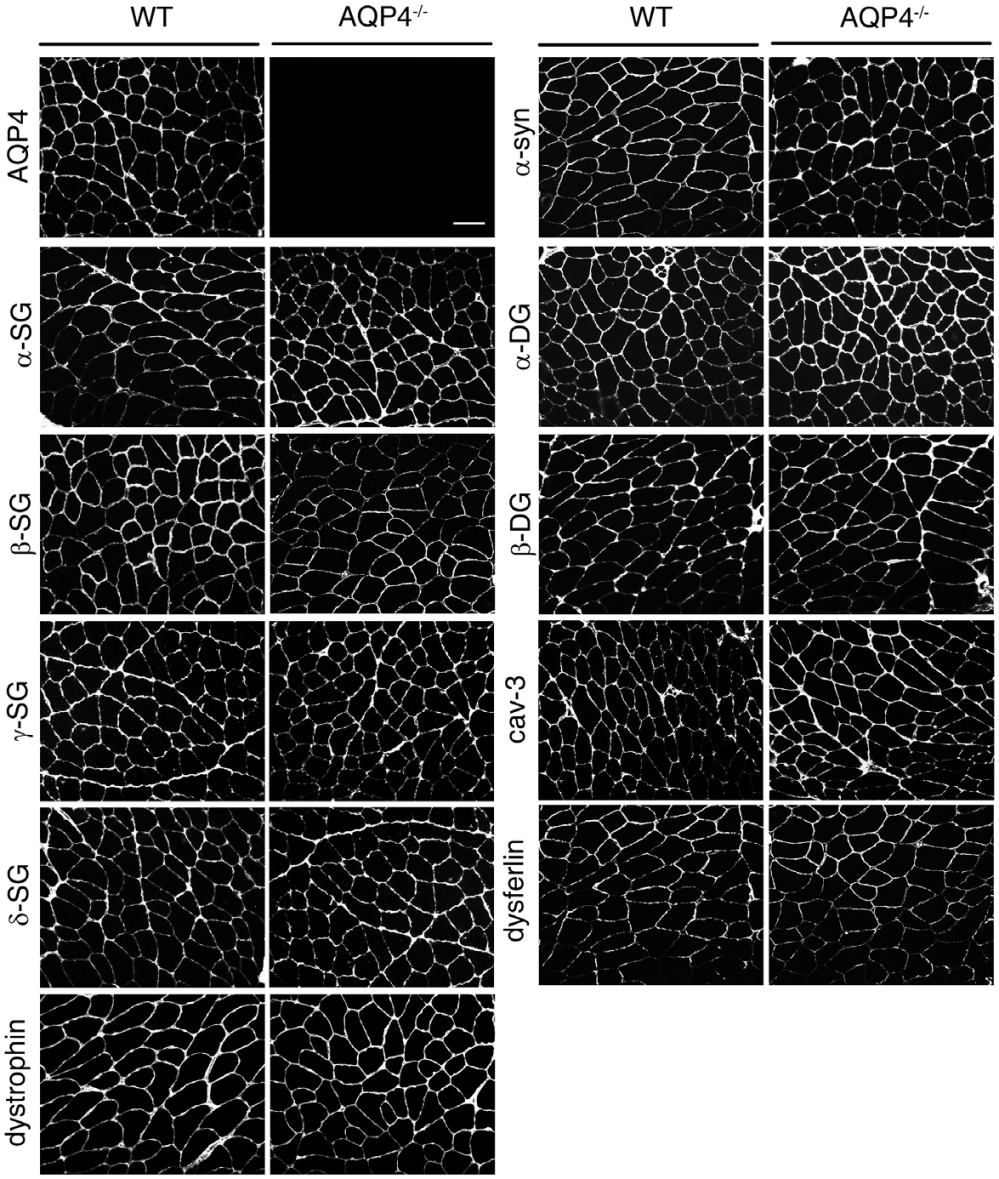
Immunofluorescent localization of AQP4 and proteins belonging to the DGC complex in WT and AQP4^−/−^ quadriceps muscle. Except for the absence of AQP4 in null mice muscle, all members of the complex are localized uniformly at the sarcolemma. Scale bar: 50 µm.

As expected, staining for AQP4 was only detected in WT muscles (Figure 1). DGC expression was robust in both WT and AQP4^−/−^ muscles: levels of SGs (α-, β-, γ- and δ-SG) and dystroglycans (α- and β-DG), as well as dystrophin, were virtually identical in terms of abundance and cellular distribution. Interestingly, α-syntrophin (α-syn), which was demonstrated to directly interact with AQP4 [15], did not show alteration in either the expression level or in sarcolemmal localization in AQP4^−/−^ muscle. Distribution of dysferlin and cav-3, two proteins of pivotal importance in membrane repair and cell signaling processes, were not affected in absence of AQP4.

Immunofluorescence results suggest that the absence of AQP4 water channels does not compromise the membrane stability associated to a defective DGC and related protein expression.

### EMC protein expression analysis by immunofluorescence

**Figure 2:**
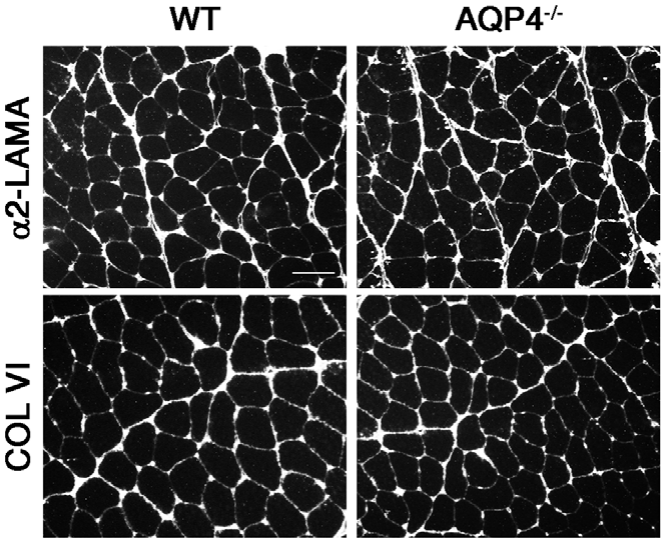
Immunofluorescence localization of EMC proteins in WT and AQP4^−/−^ quadriceps muscle. α2-laminin and collagen VI staining was regularly distributed in both WT and AQP4^−/−^ muscle fibers. Scale bar: 50 µm.

In order to determine whether AQP4 deficiency impairs ECM organization, immunofluorescence analysis was performed using antibodies against proteins belonging to the basal lamina. As shown in Figure 2, the cellular labeling of α2-laminin (α2-LAMA) and collagen VI (COLVI) was regularly distributed outside the fibers, just surrounding the basal lamina, and no differences were found between WT and AQP4^−/−^ muscles. This gives strong indications that AQP4 deficiency did not induce alteration in ECM organization.

### Sarcolemmal integrity assay

**Figure 3:**
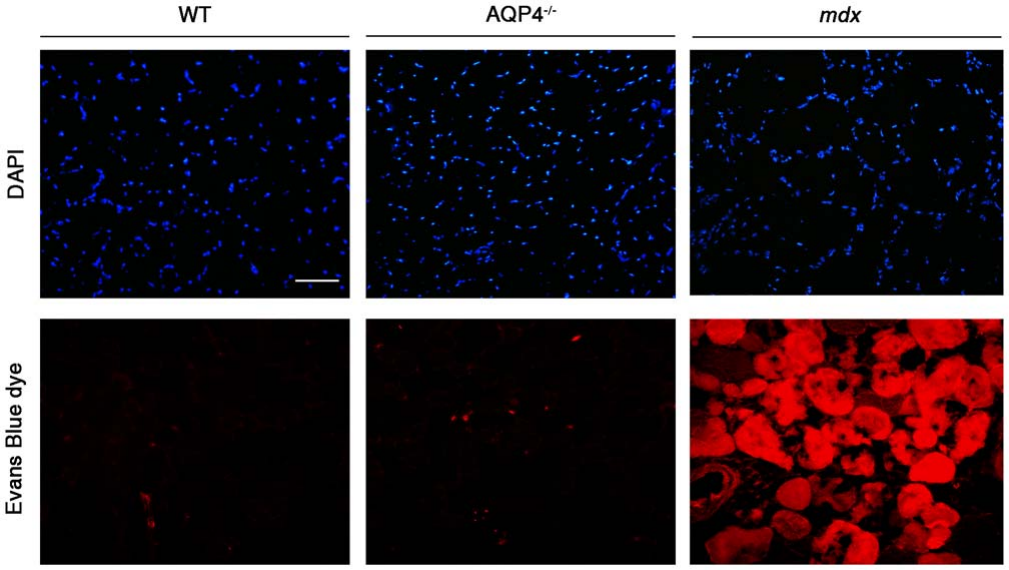
Sarcolemma integrity assay. To examine infiltration of blood serum proteins into damaged muscle fibers, WT, AQP4^−/−^ and mdx mice were intraperitoneally injected with Evans blue dye. M*dx* quadriceps displays many Evans blue dye–positive fibers (visualized by red fluorescence), due to membrane damage. Evans blue dye was minimally detected in muscle from WT and AQP4^−/−^ mice, demonstrating that the absence of AQP4 expression did not perturb sarcolemmal integrity. Scale bar: 50 µm.

Loss of the DGC from the sarcolemma causes membrane instability, which is the primary defect of dystrophin-deficient muscular dystrophy [21]–[23]. Damage to the sarcolemma allows proteins that are normally restricted to the blood serum to move across the sarcolemma and accumulate in the sarcoplasm. To further test the functionality of the DGC in AQP4^−/−^ mice and to test whether the absence of AQP4 could improve sarcolemmal stability, a tracer assay with fluorescent Evans blue dye that binds albumin in blood serum [23] was performed, using *mdx* mice as positive control (Figure 3). *Mdx* mice showed severe sarcolemmal fragility marked by Evans blue dye accumulation in numerous muscle fibers. In contrast, we never observed Evans blue–positive fibers in AQP4^−/−^ tissue nor in WT, excluding that the absence of AQP4 compromises membrane integrity.

### Comparison of 2D Blue Native (BN)/SDS-PAGE protein maps obtained from WT and AQP4^−/−^ quadriceps muscle

**Figure 4:**
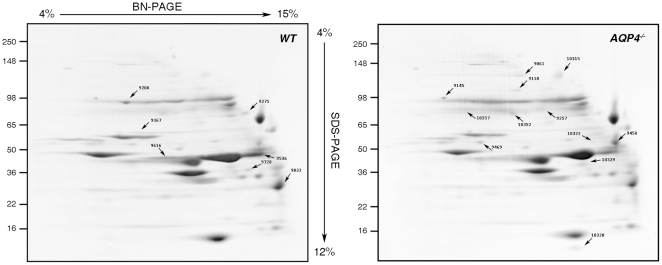
Representative 2D BN/SDS-PAGE protein pattern from WT and AQP4^−/−^ quadriceps muscle. 2D-gels were stained with Coomassie blue after a 4–15% gradient BN-PAGE and 4–12% gradient SDS-PAGE. Several protein spots were found to be altered in AQP4^−/−^ muscles. These proteins were subjected to ESI-IT and those identified are indicated with black arrows with their relative protein ID number.

To analyze differences in quadriceps muscle proteins between WT and AQP4^−/−^ mice, 2D BN/SDS-PAGE was performed. For this purpose, whole muscle proteins were extracted in BN buffer containing 1% n-dodecyl-β-D-maltoside (DDM) and subjected to 2D electrophoresis. Figure 4 shows a pair of representative 2-D gels of quadriceps muscle proteins from WT and AQP4^−/−^ mice. The WT quadriceps protein pattern was used to create a proteome master map, useful for landmarking and identifying proteins whose levels change in AQP4^−/−^ mice. Analysis performed on three triplicates of four different preparations detected more than 300 protein spots and, among them, 19 protein spots were found to be altered (*p*<0.05 significance level): 12 were found to be up-regulated in AQP4^−/−^ muscle, whereas decreases were found in expression levels of 7 protein spots. These proteins covered a broad range of molecular weights and abundance levels, and are denoted in Figure 4 by black arrows associated to their relative ID number.

### Protein identification by Mass Spectrometry (MS)

Protein identification using ESI-IT was attempted for all proteins having statistically significant changes in relative protein levels in AQP4^−/−^ quadriceps muscle. Table 1 provides protein identification for 12 protein spots whose levels of expression were up-regulated and 7 protein spots whose levels of expression were down-regulated in AQP4^−/−^ mice. MS analysis of digested spots n. 9469, n. 10315 and 10352 did not yield any reliable protein identification due to low MASCOT scores.

**Table 1:** Differentially expressed protein spots in AQP4^−/−^ quadriceps muscle compared to WT.

Spot no.	Theor. M_w_	pI predict	No. of peptides	MASCOT score	NCBI Acc. number	Protein ID
***Upregulated***						
9061	113447	5.81	9	474	gi|148690779	myosin binding protein C, fast-type, isoform CRA_a
9110	117298	6.51	4	206	gi|29145087	Oxoglutarate dehydrogenase
9145	89936	5.14	9	476	gi|55217	valosin-containing protein
9257	110723	5.13	10	651	gi|36031132	sarcoplasmic/endoplasmic reticulum calcium ATPase 1
9450	50507	8.86	6	409	gi|1583965	adenylosuccinate synthetase:ISOTYPE = muscle
9469						N.I.
10315						N.I.
10322	50764	9.11	2	110	gi|6681273	eukaryotic translation elongation factor 1 alpha 2]
10328	11937	5.02	3	183	gi|53819	parvalbumin
10352						N.I.
10357	110723	5.13	14	738	gi|36031132	sarcoplasmic/endoplasmic reticulum calcium ATPase 1
***Down-regulated***						
9200	97681	6.65	26	1564	gi|6755256	muscle glycogen phosphorylase
9275	76751	5.35	10	496	gi|148701560	Annexin A6
9367	63700	6.02	14	855	gi|33416468	Pgm2
9536	43246	6.58	14	937	gi|6671762	Creatine kinase
9616	43246	6.58	9	649	gi|6671762	Creatine kinase
9728	36045	8.44	5	330	gi|6265546	glyceraldehyde-3-phosphate dehydrogenase
9832	28980	8.65	12	748	gi|9256624	phosphoglycerate mutase 2

Only protein spots that were present on every gel (n = 4 separate comparisons) and demonstrating changes with significance p<0.05 were accepted as being differentially expressed. The proteins were identified by ESI-IT and accession numbers are given.

Interestingly, among the 13 proteins identified by MS, 7 were associated with metabolic responses of the muscle. Oxoglutarate dehydrogenase (OGDH) is a key component of the Krebs Cycle and is located in the mitochondrial matrix, where it catalyzes the decarboxylation of α-ketoglutarate to produce succinyl CoA. The expression level of OGDH increased 2.35–fold in AQP4^−/−^ quadriceps. Levels of adenylosuccinate synthetase (ADSS), which plays an important role in purine biosynthesis, by catalysing the GTP-dependent conversion of IMP and aspartic acid to AMP, increased 3.14–fold in AQP4^−/−^ quadriceps. We identified five enzymes associated with anaerobic metabolism that all showed a significant decrease in expression in AQP4^−/−^ quadriceps. The level of muscle glycogen phosphorylase (PYGM), which catalyzes the breakdown of glycogen to glucose-1-phosphate, decreased 0.43–fold in AQP4^−/−^ quadriceps. Phosphoglucomutase-2 (Pgm2), a glycolytic pathway enzyme that catalyzes the conversion of glucose-1-phosphate to glucose-6-phosphate, decreased 0.82–fold in AQP4^−/−^ quadriceps. Glyceraldheyde 3-phosphate dehydrogenase (GAPDH), which catalyzes the sixth step of glycolysis by the conversion of glyceraldehyde 3-phosphate to D-glycerate 1,3-bisphosphate, was 0.73–fold down-regulated in AQP4^−/−^ muscle. Phosphoglycerate mutase 2 (PGAM2), which catalyzes the interconversion of 2-phospho-D-glycerate and 3-phospho-D-glycerate in the eighth step of glycolisis, decreased 0.46–fold in AQP4^−/−^ muscle. The levels of two spots of muscle creatine kinase (CK-M), which reversibly catalyze the transfer of phosphate between ATP and creatine phosphate, decreased 0.26–fold (spot n. 9536) and 0.79–fold (spot n.9616) in AQP4^−/−^ muscle.

We found at least three proteins associated with calcium homeostasis that changed significantly in AQP4^−/−^ quadriceps: Annexin-6 (ANXA6), implicated in many Ca^2+^-regulated processes (e.g. vesicle trafficking, cell division, apoptosis, calcium signaling, and growth regulation), decreased 0.3-fold in AQP4^−/−^ muscle. Levels of parvalbumin (PV), a small calcium binding protein strongly involved in muscle relaxation, increased 3.1–fold in AQP4^−/−^ muscle. Sarcoplasmic/endoplasmic reticulum calcium ATPase 1 (SERCA1), which is an ATP-dependent calcium pump responsible, in part, for the maintenance of low cytoplasmic free calcium concentrations, was found to be upregulated in two spots: the spot n. 9257 increased 4.48-fold, and the spot n. 10357 increased 2.74-fold.

Among the advantages of BN/SDS-PAGE, this particular technique can provide rapid information on the number, size, composition, and relative abundance of complexes that a given protein forms. Interestingly, the spots corresponding to SERCA1 were horizontally aligned and this means that it is organized in at least two multiprotein complexes (MPCs) of different size.

Levels of myosin binding protein C, fast-type 2 (MYBP-C2), a thick filament-associated protein that modulates muscle contraction, increased 3.6-fold in AQP4^−/−^ quadriceps. Valosin-containing protein (VCP) is implicated in multiple cellular processes, including cell cycle regulation, nuclear envelope formation, Golgi biogenesis, and the ubiquitin proteasome system [24]. This protein was found to be 2.53-fold upregulated in AQP4^−/−^ quadriceps. Eukaryotic translation elongation factor 1α2 (eEF1A2), whose canonical function is translational elongation, is normally expressed only in muscle, brain, and heart. The levels of this protein increased 3.31-fold in AQP4^−/−^ muscle.

### In silico elaboration of proteomics data: protein-protein interaction and Gene Ontology (GO) term enrichment

**Figure 5:**
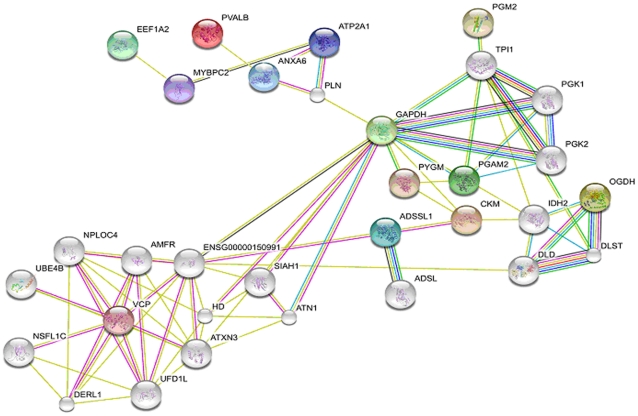
Protein-protein interaction map of the MS-identified proteins. Protein IDs obtained upon MS-based identification of spots of interest were uploaded to String 8.3 for protein-protein interaction mapping. High degree nodes (GAPDH and VCP) and three main clusters of proteins (right, bottom-left, upper-left) were individuated.

Proteins identified through MS were elaborated *in silico* for the identification of likely networks of proteins which proved to be differentially expressed in the experimental model under analysis. Criteria for the interpretation of protein-protein interaction maps include the individuation of the centrality (position in the map) and degree of the nodes. The degree of a node indicates the number of links connected to a vertex. High degree nodes are the most relevant ones in interactome networks, as they represent the fulcrum of multiple signaling pathways, and their positive/negative modulation could result in alterations of the activities of their likely interactors. High degree nodes deriving from *in silico* elaboration of “omic” data (i.e. proteomics) are thus best suited for subsequent targeted approaches (e.g. western blot analysis). Upon interrogation of the String database, the protein-protein interaction map obtained from the analysis (Figure 5) showed a central high degree node accounting for the glycolytic enzyme GAPDH and VCP. Three main clusters (groups of highly-interacting proteins) could be observed, including a group of metabolism-related proteins in the right portion of the map, skeletal muscle contraction (upper-left) and ubiquitination (bottom-left). To determine whether functional classification could be refined and expanded, FatiGO/Babelomics was used to perform GO term enrichment for biological and molecular functions and sub-cellular compartment localization (Table 2).

**Table 2:** GO term enrichment.

#Index	Term	#1 vs #2	P-value	Pivotal Proteins
**Molecular function**				
1	intramolecular transferase activity (GO:0016866, GO:0016772)	40% vs 0.12%	0.007467	CKM, PGM2, PGAM2,
3	calcium ion binding (GO:0005509)	40% vs 0.34%	0.008142	PV, ANXA6, ATP2A1
2	cofactor binding (GO:0048037)	30.77% vs 1.79%	0.002451	GAPDH, OGDH, PYGM, PGAM2
**Biological function**				
1	monosaccharide metabolic process (GO:0005996)	60% vs 1.02%	0.007606	PGM2,GAPDH,PGAM
2	glucose metabolic process (GO:0006006)	60% vs 0.71%	0.007171	PGM2, GAPDH, PGAM2, PYGM
**Cellular component**				
1	sarcoplasm and myofibril (GO:0016528, GO:0030016)	25% vs 0.17%	0.00883	ATP2A1, PYGM; MYBPC2
2	sarcoplasmic reticulum (GO:0016529)	25% vs 0.16%	0.00883	ATP2A1, PYGM

Significantly (p-value<0.05) up-regulated ontologies (*vs* the rest of the mouse genome) are reported.

Although the list of identified proteins only includes a limited number of entries, GO term enrichment helped elucidate whether one specific category of biological and/or molecular function was particularly altered in the experimental model under analysis (Table 2). The most significant results were obtained for biological functions, which highlighted the presence of a group of proteins involved in monosaccharide metabolic process (GO:0005996 - PGM2,GAPDH,PGAM) and glucose metabolic process (GO:0006006 - PGM2, GAPDH, PGAM2). As for molecular functions, proteins identified as being differentially expressed mainly accounted for phosphotransferase activities (intramolecular transferase activity (GO:0016866, GO:0016772 – CKM, PGM2, PGAM2), cofactor binding (GO:0048037 - GAPDH, OGDH, PYGM, PGAM2) and calcium ion binding (GO: 0005509 - PV, ATP2A1), the latter group being particularly relevant in muscle relaxation/contraction. Determination of GO for cellular component localization yielded quite predictable results, indicating muscle-specific localization for most of the proteins of interest: sarcoplasm and myofibril (GO:0016528, GO:0030016); sarcoplasmic reticulum (GO:0016529).

### Proteomic validation by immunoblot analysis

2-D immunoblotting with highly specific primary antibodies was employed to confirm altered expression levels of distinct quadriceps muscle proteins. Proteins were electrophoretically separated as described above and then transferred onto PVDF membrane. Target proteins were chosen upon evaluation of the proteomics analysis (differential quantitation) and *in silico* elaboration of the data, through the consideration of a series of criteria including high degree of nodes, centrality in the groups emerging from the protein-protein interaction analysis and most relevant biological/molecular functions. For this purpose, two up-regulated (PV and VCP) and two down-regulated proteins (GAPDH and CK-M) in AQP4^−/−^ muscle were chosen.

**Figure 6:**
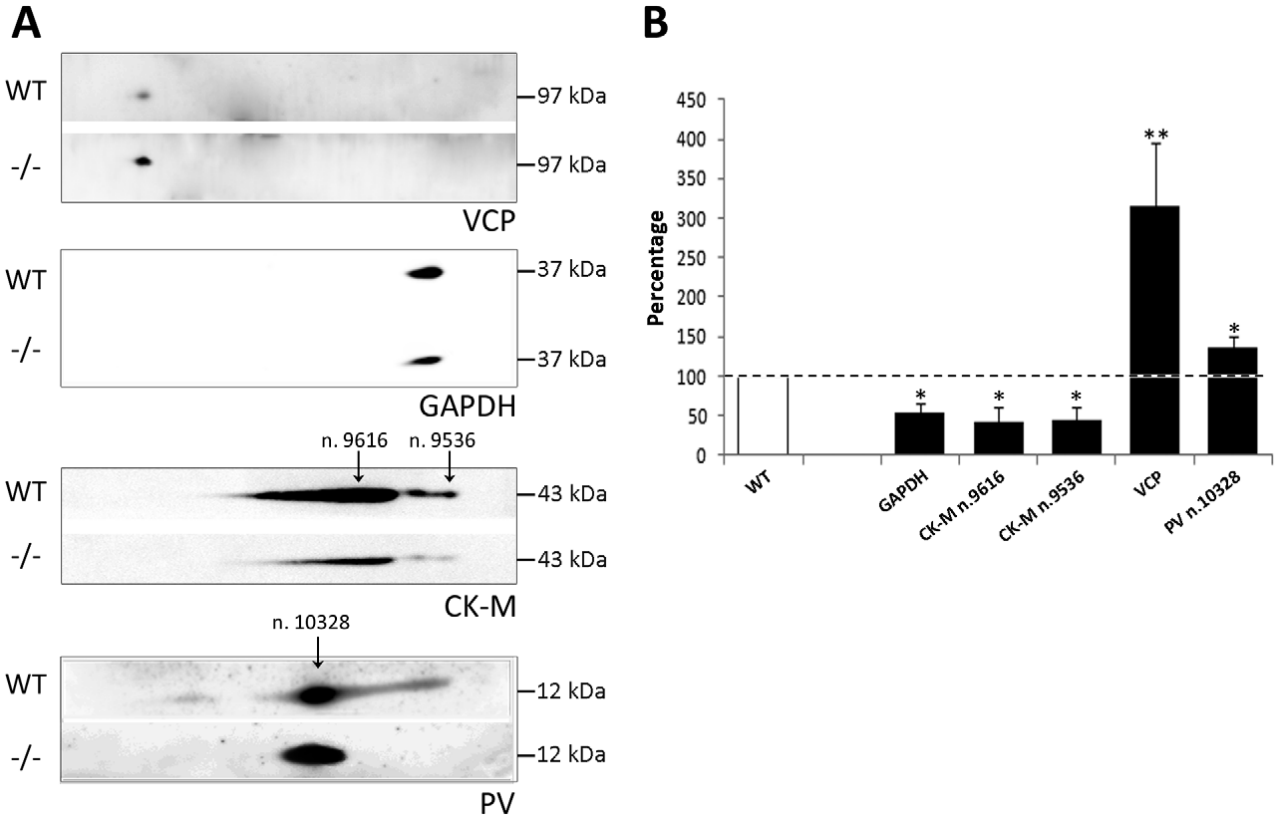
Immunoblotting analysis of proteins altered in AQP4^−/−^ muscle. A) Proteins were separated by 2D BN/SDS-PAGE and immunoblotted using antibodies against the indicated proteins. B) Semi-quantitative densitometric analysis of expression levels of VCP, GAPDH, CK-M and PV. Note that all alterations had a high statistical significance. (*p<0.05; **p<0.01).

Figure 6 shows the immunoblot results of the above mentioned proteins, along with a semiquantitative analysis. Concerning core nodes from Figure 5, results of VCP and GAPDH expression levels in AQP4^−/−^ muscles confirmed the alterations observed by proteomic analysis, with a 3-fold increase and a 0.48-fold decrease respectively.

Among the protein spots that were selected for identification, two corresponded to CK-M and were found to be down-regulated. Immunoblot analysis revealed that CK-M was organized in three MPCs, all down-regulated in AQP4^−/−^ muscle. The signals corresponding to spots n. 9536 and n.9616 were found to be about 0.5-fold decreased compared to WT.

The immunoblot result of PV expression was of particular interest: this protein was found to be up-regulated in AQP4^−/−^ quadriceps muscle, and the relative spot in the 2-DE map, corresponding to n. 10328 (black arrow in Figure 6A), increased the abundance level by 0.3-fold compared to WT. Interestingly, the antibody reaction in WT muscles revealed the expression of PV in approximately three different MPCs, whereas only one of them was present in AQP4^−/−^ muscles. These results suggest that in WT muscle multiple PV complexes may exist possibly located in different areas of the fibers.

We can conclude that the analysis of the proteome of the AQP4^−/−^ quadriceps muscle showing differential expression of protein was correctly portrayed.

## Discussion

The aim of this study was to analyze to what extent the absence of AQP4 in the sarcolemma affects muscle protein expression and sarcolemma integrity.

Immunofluorescence data obtained in quadriceps muscles suggest that the absence of AQP4 does not impair the expression of the proteins belonging to DGC or their cellular distribution in myofibers. AQP4 deletion does not alter the expression of the most important components of ECM. Furthermore, Evans blue analysis allowed us to determine the absence of major alterations in sarcolemma integrity in AQP4^−/−^ skeletal muscle. Taken together, these data suggest that the absence of AQP4 in skeletal muscle does not alter the DGC-ECM scaffold composition of the sarcolemma and does not result in a dystrophic phenotype. This also indicates that the alteration of AQP4 expression observed in several forms of muscular dystrophy are probably not a consequence of a direct structural interaction of the water channel with DGC proteins. However, we cannot exclude a functional interaction that could be influenced by fiber metabolism.

We then decided to conduct a proteomic investigation of the effects of the absence of AQP4 in skeletal muscle with the aim of determining a possible altered protein expression pattern using techniques of BN/SDS-PAGE and peptide mass fingerprinting. BN/SDS-PAGE allows effective separation of protein species and complexes from various subcellular origin preparations. In particular, this technique seems particularly well suited for separation of transmembrane proteins and proteins with high isoelectric point values that usually fail to enter the first dimension of 2D IEF/SDS gels. In fact, among the proteins identified by MS, four spots correspond to membrane proteins and four proteins have an isoelectric point value higher than 8.5, probably rendering their separation by the usual 2D gel separation method difficult or impossible. These findings allow us to conclude that 2D BN/SDS-PAGE constitutes an interesting alternative to conventional two-dimensional electrophoresis, in agreement with recent literature [25].

By this approach, we found that the most abundant group of altered proteins in AQP4^−/−^ mice is represented by proteins involved in metabolism (Table 2). Interestingly, down-regulation of glycolytic enzymes (PYGM, Pgm2, GAPDH and PGAM2 - GO:0006006) and phosphagen regulatory enzymes (CK-M - GO:0016772) suggests that there is a strong glycemic imbalance and, in general, alterations in bioenergetic pathways in AQP4^−/−^ muscle. This latter consideration is supported by the increase in OGDH, the cofactor binding (GO:0048037), in a key enzyme of oxidative metabolism (GO:0004591) in the Krebs cycle, and in ADSS, the activity of which is also related to ATP synthesis through purine metabolism, probably to compensate energy supply by glycolysis. The production of 2 mol of ATP from 1 mol of metabolized glucose makes glycolysis a less efficient biochemical pathway as compared to oxidative phosphorylation, but the high abundance of fast-acting glycolytic enzymes nevertheless provides a substantial amount of energy for skeletal muscle contraction. Glycolysis is an absolutely essential metabolic system during both anaerobic and aerobic conditions. Hence, the high concentration and the central metabolic position make expression levels of glycolytic enzymes susceptible to drastic changes during physiological adaptations or pathological alterations, such those occurring in several myopathies [26] and in disuse-induced muscle atrophy [27], [28], in which a strong rearrangement of AQP4 expression is also found. This gives further support to our original idea [2] that the expression of AQP4 is associated with the glycolitic metabolism of the fiber and that its functional role may be linked to response to physiological needs during muscle fatigue, probably in the regulation of water homeostasis in response to metabolite production during muscle contraction. This hypothesis is markedly supported by our preliminary observation in mice lacking AQP4, showing impaired activity of skeletal muscle when subjected to both voluntary and endurance exercises [29].

A set of proteins altered in AQP4^−/−^ mice is involved in calcium homeostasis. In particular, ANXA6 is closely associated in the selective modulation of the activity of the Ca^2+^ channel involved in its release from sarcoplasmic reticulum (calcium ion binding - GO: 0005509 in Table 2) [30]. Interestingly, ANXA6 down-regulation is accompanied by an increase in SERCA1 expression levels, which is probably related to the modulation of the events involved in excitation-contraction coupling (regulation of muscle contraction – GO:0006937). In addition, up-regulation in a specific MPC of PV expression in AQP4^−/−^ mice confirms that alteration in Ca^2+^ handling occurs, since PV is the most important soluble protein involved in Ca^2+^ buffering during muscle relaxation. It is worth noting that a qualitative difference in PV expression levels between WT and AQP4^−/−^ mice is observed, suggesting that a rearrangement in subcellular organization probably occurs without significant variation in total amount of protein. It cannot be ascertain whether alterations in expression levels of proteins described above are directly affecting muscle contraction, even if MYBP-C2 (myofibril - GO:0030016), which is a protein belonging to contractile apparatus, was found to be up-regulated in AQP4^−/−^ mice. The altered expression of this protein could be a response to variations in Ca^2+^ buffering. Therefore, a new function of AQP4 is probably related to its action on intracellular Ca^2+^ dynamics, as also suggested by altered spontaneous Ca^2+^ oscillations observed in ANSCs from AQP4^−/−^ mice [20], and more recently, in astrocytic Ca^2+^ signaling events elicited by cerebral edema [31]. Moreover, it is interesting that alterations found in AQP4^−/−^ muscle affected proteins is typically expressed in skeletal muscle fast-twitch fibers, the same in which AQP4 is abundantly expressed.

The translational elongation factor eEF1A2 is a guanine-nucleotide binding protein, which transports aminoacylated tRNA to the ribosomal A site during protein synthesis. Recent evidences suggest an important role for eEF1A2 in driving cap-independent translation of utrophin A in skeletal muscle [32]. Moreover, a specific interaction with a novel myosin binding protein-C-like protein has been described [33], suggesting that increased expression in eEF1A2 in AQP4^−/−^ mice might be related to that observed for MYBP-C2.

VCP is an ATPase involved in a number of cellular activities, including homotypic membrane fusion, transcription activation, nuclear envelope reconstruction, post-mitotic organelle reassembly, cell cycle control, apoptosis and endoplasmic reticulum associated degradation of proteins [34]–[37]. The latter function resulted in a cluster of interacting proteins which can be observed in the bottom-left portion of the map in Figure 5. Although the great number of functions in which this protein could be involved make it difficult to rule out a unique effect of the observed VCP increase in AQP4^−/−^ muscle, the protein-protein interaction map seems to suggest a particular role of VCP over-expression in the up-modulation of protein-ubiquitination homeostasis. Recent analyses on tissues from patients suffering from inclusion body myopathy (IBM) associated with Paget's disease of the bone and fronto-temporal dementia (PFD) appear to support this hypothesis [38]. Indeed, it has been demonstrated that over-expression of two mutated isoforms of VCP lead to the accumulation of ubiquitinated body inclusions and protein aggregates in patient tissues. This results in muscle weakness, which is the presenting symptom in more than half of IBM/PFD patients and an isolated symptom in 30% of the cases of these multi-system disorders [39]. However, the consequences of normal VCP over-expression in skeletal muscles are yet to be fully explored.

In conclusion, no major alterations have been found in DGC, ECM or sarcolemma integrity in AQP4^−/−^ mice as could have been supposed to occur. Instead, examining expression levels of structurally or functionally related proteins on a large scale BN/SDS-PAGE coupled with other biochemical approaches has provided us with information on which proteins are responsive to AQP4 deficiency. This study demonstrates that the absence of AQP4 in skeletal muscle has effects on regulatory trends of bioenergetic pathways and Ca^2+^ handling, without perturbing the integrity of the sarcolemma, This establishes an important novel role of AQP4 in the skeletal muscle and gives new evidence about the relationship between AQP4 expression and skeletal muscle metabolism, as previously hypothesized by our group [2].

## Materials and Methods

### Ethics statement

All experiments conformed to international guidelines on the ethical use of animals and were designed to minimize the number of animals used and their suffering. Experiments in this study were approved by the Italian Health Department (Art. 9 del Decreto Legislativo 116/92).

### AQP4^−/−^ transgenic mice

AQP4^−/−^ mice with a CD1 genetic background were kindly provided by Dr. Hu (Nanjing Medical University, China). The generation of this AQP4 null mice model has been previously reported [16]. The mice used here were bred and genotyped in an approved facility at the University of Bari. Mice were kept on a 12 h light-dark cycle with food and water ad libitum. Male WT and AQP4^−/−^ mice with a CD1 genetic background, aged 3 months, were used in these experiments.

### Chemicals

ε-Aminocaproic acid, Imidazole, Tricine, and Ferritin were from Fluka (St Louis, MO, USA). Acrylamide/bisacrylamide was from Serva (Heidelberg, Germany). Protease inhibitor cocktail was purchased from Roche Diagnostics (Indianapolis, IN, USA). All other chemicals were obtained from Sigma (St Louis, MO, USA).

### Antibodies

Rabbit polyclonal antibody against α-syn has been previously characterized [40]. Rabbit polyclonal anti-PV antibody was purchased from Synaptic System (Gottingen, Germany). Goat polyclonal anti-AQP4 and CK-M, rabbit polyclonal anti-VCP and anti-β-DG, mouse monoclonal anti-cav-3 and anti-α2-LAMA antibodies were purchased from Santa Cruz Biotechnology (Santa Cruz, CA, USA). Mouse monoclonal anti-α-DG antibody was purchased from Upstate Signaling (Lake Placid, USA). Mouse monoclonal anti-α-, β-, γ- and δ- SGs, and anti-dysferlin antibodies were purchased from Novocastra (Newcastle-upon-Tyne, UK). Mouse monoclonal anti-GAPDH antibody was purchased from Millipore (Billerica, MA, USA). Mouse monoclonal anti-dystrophin (MANDYS1) was purchased from Developmental Studies Hybridoma Bank. Rabbit polyclonal anti-COLVI antibody was purchased from Abcam (Cambridge, MA, USA).

### Immunofluorescence

Quadriceps from male CD1 AQP4^−/−^ and WT littermates were dissected from 3-month-old mice. Muscles were rapidly frozen in liquid nitrogen–cooled isopentane. 8-µm transverse sections were prepared using a cryostat (CM 1900; Leica) and stored on positively charged glass slides (Thermo Scientific). Sections were acclimated to RT for 15 min and blocked using 0.1% gelatin diluted in PBS for 30 min at RT. Sections were then incubated at RT for 1 h with antibodies to the following proteins (antibody dilutions are indicated): AQP4 (1∶300), dystrophin (1∶10), α-DG (1∶100), β-DG (1∶300), α-SG (1∶50), β-SG (1∶100), γ-SG (1∶50), δ-SG (1∶50), cav-3 (1∶200), dysferlin (1∶100), α-syn (1∶1000), α2-LAMA (1∶500), COL VI (1∶200). Primary antibodies were detected by AlexaFluor 488 and 594 anti–rabbit, anti-goat and anti–mouse (Invitrogen, CA, USA) secondary antibodies diluted at 1∶1000. Secondary antibodies were incubated for 1 h at RT. To preserve the fluorescence signal, sections were mounted in a medium containing DAPI. The sections were examined with a Nikon photomicroscope equipped for epifluorescence (DMRXA; Leica, Heidelberg GmbH, Mannheim, Germany). Digital images were obtained with a DMX 1200 camera (Nikon, Tokyo, Japan).

### Evans blue tracer assay

To test sarcolemmal integrity, mice were injected with 50 µl of Evans blue dye (10 mg/ml in 10 mM of sterile phosphate buffer and 150 mM NaCl, pH 7.4) per 10 g of body weight [23]. Peritoneal cavity injection was performed on 3-month-old AQP4^−/−^, *mdx* and relative WT mice. 24 h after injection, quadricepses were excised and mounted as described in the “Immunofluorescence” section. 8-µm transverse sections were briefly fixed in ice-cold acetone, washed in PBS (10 mM phosphate buffer and 150 mM NaCl, pH 7.4), and mounted with a medium containing DAPI. Evans blue–positive myofibers were observed with a Leica DMRXA photomicroscope equipped for epifluorescence, and digital images were obtained as previously described.

### Sample preparation

Skeletal muscle was snap frozen in liquid nitrogen and ground to a fine powder with a mortar and pestle. Pulverized muscle was added to ice-cold BN Lysis Buffer (ε-aminocaproic acid 500 mM, Imidazole 7.5 mM, EDTA 2 mM, NaCl 12 mM, glycerol 10%, DDM 1%, PMSF 1 mM) and protease inhibitor cocktail (Roche). The lysis was performed on ice for 1 h and the samples were then centrifuged at 22,000×g for 1 h. The protein content of the supernatant was measured with BCA Protein Assay Kit (BioRad, Rockford, IL, USA).

### 2D BN/SDS-PAGE

Two-dimensional BN/SDS-PAGE is a particular electrophoretic technique for the global analysis of the subunits of complexes in proteomes [41]. In the first non-denaturing dimension (BN-PAGE) complexes ranging from few kDa to several MDa are separated, and their subunits are resolved in the second denaturing dimension (SDS-PAGE). With this method we are able to detect proteins with molecular masses between 10 kDa and up to 200 kDa. 2D BN/SDS-PAGE on skeletal muscle proteins was performed as previously described [6] with some modifications. Briefly, 250 µg of protein sample was applied on 4–15% polyacrylamide native gradient gels. The running buffers were the anode buffer (25 mM Imidazole, pH 7) and the blue cathode buffer (7.5 mM Imidazole, pH 7, 50 mM Tricine, and 0.02% CBB G-250). Electrophoresis was performed at 10 mA for approximately 8 h at 10°C, and stopped when the tracking line of CBB G-250 dye had left the edge of the gel. The lanes from the first dimension were cut into individual strips and, if necessary, frozen at −20°C. The strips were equilibrated in denaturing buffer (1% SDS and 1% β-mercaptoethanol) for 30 min at room temperature and placed into a 2D SDS-PAGE of the same thickness. The second-dimensional separation was performed on 4–12% SDS-polyacrylamide gels and conducted at 50 mA per gel. At the end of the run, the gel was either Coomassie-stained or blotted onto a PVDF membrane (Millipore) for Western blot analysis.

### Gel staining and image analysis

The gels were washed in ultrapure distilled water several times, then incubated overnight in 0.12% colloidal CBB G-250 solution [42], washed again with ultrapure distilled water until the blue background disappears. Each stained slab gel was digitized and processed using the ImageMaster 2D 6.0 software system (GE Healthcare) to yield a spotlist giving position, shape and density information for each detected spot. Scanned gel images (three replicates of each sample) were then analyzed and each 2-D gel was matched with an appropriate “master” 2-D gel.

### In-Gel Digestion

Spots from 2-DE maps of biological interest (P<0.05) were carefully excised from the gel and subjected to in-gel trypsin digestion according to Shevchenko et al. [43] with minor modifications. The gel pieces were swollen in a digestion buffer containing 50 mM NH_4_HCO_3_ and 12.5 ng/mL trypsin (modified porcine trypsin, sequencing grade, Promega, Madison, WI) in an ice bath. After 30 min, the supernatant was removed and discarded; then 20 µL of 50 mM NH_4_HCO_3_ was added to the gel pieces, and digestion was allowed to proceed overnight at 37°C. The supernatant containing tryptic peptides was dried by vacuum centrifugation. Prior to mass spectrometric analysis, the peptide mixtures were redissolved in 10 µL of 5% formic acid (FA).

### Protein identification by Nano-RP-HPLC-ESI-MS/MS

MS procedures were performed as previously described [44]. Peptide mixtures were separated using nanoflow-HPLC system (Ultimate; Switchos; Famos; LC Packings, Amsterdam, The Netherlands). A sample volume of 10 µL was loaded by the autosampler onto a homemade 2 cm fused silica pre-column (75 µm I.D.; 375 µm O.D) Reprosil C18-AQ, 3 µm (Ammerbuch-Entringen, DE) at a flow rate of 2 µL/min. Sequential elution of peptides was accomplished using a flow rate of 200 nL/min and a linear gradient from Solution A (2% acetonitrile; 0.1% FA) to 50% of Solution B (98% acetonitrile; 0.1% FA) in 40 min over the precolumn in-line with a homemade 10–15 cm resolving column (75 µm I.D.; 375 µm O.D.; Reprosil C18-AQ, 3 µm (Dr. Maisch GmbH, Ammerbuch-Entringen, Germany). Peptides were eluted directly into a High Capacity ion Trap HCTplus (Bruker-Daltonik, Bremen, Germany). Capillary voltage of 1.5–2 kV and a dry gas flow rate of 10 L/min were used at a temperature of 200°C. The scan range used was from 300 to 1800 m/z. Protein identification was performed by searching in the National Center for Biotechnology Information non-redundant database (NCBInr, version 20081128, www.ncbi.nlm.nih.gov) using the Mascot program in-house version 2.2 (Matrix Science, London, UK). The following parameters were adopted for database searches: taxonomy Mammals; complete carbamidomethylation of cysteines and partial oxidation of methionines, peptide Mass Tolerance ±1.2 Da, Fragment Mass Tolerance ±0.9 Da, missed cleavages 2. For positive identification, the score of the result of (−10×Log(P)) had to be over the significance threshold level (P<0.05). Even though high MASCOT scores are obtained with values greater than 60, when proteins were identified by one peptide only a combination of automated database search and manual interpretation of peptide fragmentation spectra was used to validate protein assignments. In this manual verification the mass error, the presence of fragment ion series and the expected prevalence of C-terminus containing ions (Y-type) in the high mass range were all taken into account. Moreover, replicate measurements confirmed the identity of the protein hits.

### Protein-protein interaction analysis

Proteins in biological systems seldom work as independent entities, rather they are placed in widely interacting regulatory networks. Therefore, the study of protein-protein interactions is a valid instrument to retrieve biologically relevant insights, through the individuation of pivotal webs of proteins and their likely main modulators. Network analysis software, such as String, determines and makes graphs of unbiased networks, in which gene products are represented as nodes, and the biological relationship between two nodes is represented as an edge (line). All edges are supported by at least one reference from the literature, from a textbook, or from canonical information stored in the software internal database. STRING 8.3 software [45] was used to perform functional annotation through mapping protein interactors to the experimentally identified protein species. Proteins identified experimentally were updated in the software along with indications of the proteomic analysis and the species under investigation in order to exclude false-positive protein-protein interactions and functional annotations derived from investigations on other species. An internal algorithm identifies proteins from the submitted list in the STRING database and maps them as grey nodes. White nodes represent predicted interactors upon matching against the internal database. The confidence interval was set to 0.700 (high confidence), additional white nodes to 20 and network depth was kept to the minimum value (1), in order to exclude as many false positive interactions as possible.

### GO term enrichment for biological and molecular functions and cellular component localization

GO term enrichment for biological functions was performed as previously reported [46], in order to determine whether the observed inter-group variations affected a functional cluster of proteins sharing similar biological and molecular functions, or co-localizing in a sub-cellular compartment. Protein lists were uploaded to FatiGO/Babelomics 4 [47] software for comparison of the experimentally obtained dataset against the whole murine genome. Upon interrogation of the internal database, the software converted protein entries into a list of GO terms indicating protein biological and molecular functions, as well as physiological cell localization, using the corresponding gene-GO association table. Then, a Fisher's exact test was used to check for significant over-representation of GO terms in the submitted dataset against the remaining ones from the *Mus musculus* genome.

### Western blot analysis

Equal amounts of protein sample were separated by BN/SDS–PAGE performed as described previously. Membranes with blotted proteins were incubated with the following primary antibodies: PV (1∶2000), VCP (1∶500), GAPDH (1∶2000), CK-M (1∶1000). After being washed, membranes were incubated with anti-mouse, anti-goat and anti-rabbit peroxidase-conjugated secondary antibodies (Santa Cruz) at a dilution of 1∶10000 and washed again. Reactive proteins were revealed with an enhanced chemiluminescent detection system (ECL Plus; GE Healthcare, Buckinghamshire, UK) and visualized on a Versadoc imaging system (BioRad). Densitometric analysis was performed using Scion Image software (Frederick, MD, USA).

### Statistics

Statistically significant differences were computed using the Student's *t*-test, the significance level being set at p<0.05. Only proteins with spot volumes consistently different in all replicate gels were considered differentially expressed.
